# Traceability of Sustainability and Safety in Fishery Supply Chain Management Systems Using Radio Frequency Identification Technology

**DOI:** 10.3390/foods10102265

**Published:** 2021-09-24

**Authors:** Labonnah Farzana Rahman, Lubna Alam, Mohammad Marufuzzaman, Ussif Rashid Sumaila

**Affiliations:** 1 Institute for Environment and Development, Universiti Kebangsaan Malaysia, Bangi 43600, Selangor, Malaysia; labonnah.deep@gmail.com (L.F.R.); r.sumaila@oceans.ubc.ca (U.R.S.); 2 Institute of Energy Infrastructure, Universiti Tenaga Nasional, Kajang 43000, Selangor, Malaysia; marufsust@gmail.com; 3 Faculty of Science, Institute for the Oceans and Fisheries, The University of British Columbia, Vancouver, BC V6T 1Z4, Canada

**Keywords:** food safety, sustainability, RFID, technology, fisheries, consumption, traceability

## Abstract

At present, sustainability and emerging technology are the main issues in any supply chain management (SCM) sector. At the same time, the ongoing pandemic is increasing consumers’ concerns about food safety, processing, and distribution, which should meet sustainability requirements. Thus, supervision and monitoring of product quality with symmetric information traceability are important in fresh food and fishery SCM. Food safety and traceability systems based on blockchain, Internet of Things (IoT), wireless sensor networks (WSN), and radio frequency identification (RFID) provide reliability from production to consumption. This review focuses on RFID-based traceability systems in fisheries’ SCM, which have been employed globally to ensure fish quality and security, and summarizes their advantages in real-time applications. The results of this study will help future researchers to improve consumers’ trust in fisheries SCM. Thus, this review aims to provide guidelines and solutions for enhancing the reliability of RFID-based traceability in food SCM systems so to ensure the integrity and transparency of product information.

## 1. Introduction

In this pandemic era, traceability is a vital safety tool for food supply chain management (SCM) systems, especially for fresh food and live products. The increasing demand of a healthy lifestyle by consumers makes it necessary to trace the quality and security of food products [1]. Due to the pandemic, aquaculture and capture fisheries have largely expanded the controls to ensure food securities. At present, in many countries, people are subjected to movement restrictions, as the zoonotic disease COVID-19 (caused by SARS-CoV-2) is highly contagious [2]. Moreover, because of the COVID-19 pandemic, people are paying more attention to high-quality, secure, and traceable fresh foods, seafood, medicines, etc. [3]. However, product information asymmetry and food contamination are decreasing consumers’ trust in the market. As a result, logistic chain industries are facing significant losses due to disrupted fish production. Additionally, clients’ awareness of safety requirements with regard to fish/seafood consumption has changed [2]. Consequently, supervision and monitoring of product safety, besides its quality, impose traceability features from production to distribution. Many countries have developed and depend on online systems to trace and assess the quality of fresh food in their SCM systems to reduce product information asymmetry and related ethical issues and constantly enhance food value [3,4].

There has been a rapid growth of technologies and wireless communication systems such as blockchain, Internet of Things (IoT), wireless sensor networks (WSN), and radio frequency identification (RFID) to trace and assess the safety of fresh food or processed food in the SCM process. The IoT and WSN mainly focused on smart sensing and communication whereas the blockchain is focused on assessing the data and prevent concurrent transactions. Among those technologies, RFID is widely used technology with mobility, inventory accuracy, and secure traceability features. Moreover, real-time information can be accessed by consumers through RFID using the IoT solution [5,6,7]. Additionally, RFID-based traceability in SCM increases asset visibility, expands employee productivity, and mitigates risk and the theft or loss of products. Consequently, the RFID system appears ideal for retailers and consumers in the SCM system to monitor fresh food production, fishing industries, and the aquaculture sector [6,8,9]. At present, the manufacturing/production process uses the RFID technology thanks to its effectiveness and profitability. Additionally, it allows the public to obtain the track records/footprints of foodstuffs and, through the acquired information, participate in the entire SCM process [10,11,12,13]. As COVID-19 imposes social distancing and requires a reduction in field workers and administrative staff, the implementation of RFID-based SCM and distribution networks has been studied for the agri-food and fishery sectors, warehouse management, hospital management, and SCM [14,15,16,17]. Research has been conducted on implementing the RFID technology in supply chains, including fresh food SCM, order management, inventory system, and the aquaculture sector [18,19].

**Figure 1:**
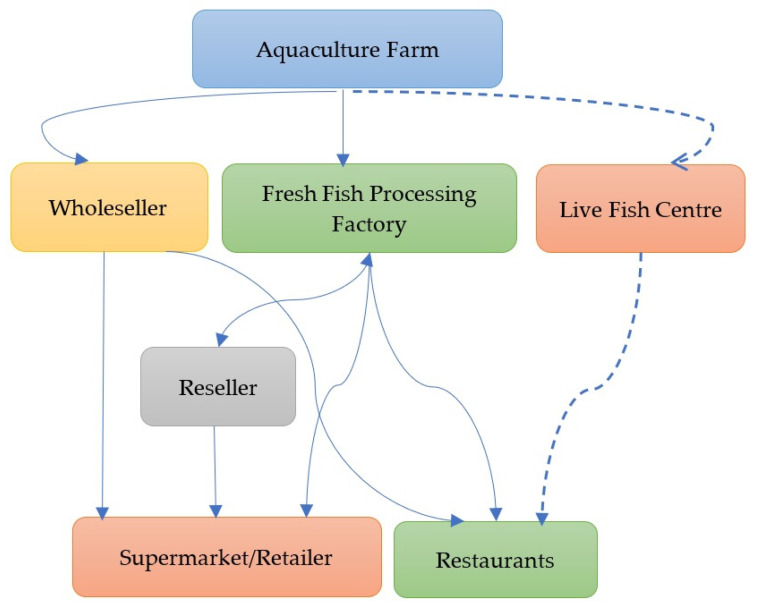
Conceptual diagram of the aquaculture SCM system.

Food traceability has become a priority worldwide in the food SCM system to de-crease consumers’ perceived food risks and raise their trust. This is related to the concept of tracking a product—food in general, including fish and fish products—from the farm to the table [20,21,22,23,24]. As a result, new regulations are necessary to ensure food safety and, therefore, new stringent food safety and trade policies [21]. As fresh fish is also a perishable product, a fishery SCM system should strictly monitor the environmental parameters in every step. The seafood SCM contains eight important phases, with source; hatchery operations (aquaculture only); nursery operations (aquaculture only); on growing techniques/wild; harvesting; processing; market, and consumers [23]. Figure 1 shows the conceptual framework for the aquaculture SCM process.

The fishery supply chain is long and complex, and its multiple components are difficult to manage and trace. Additionally, different fish industries have different production and distribution chains depending on the product (e.g., a cold chain should be maintained for tuna fish) [22]. On the other hand, a seafood supply chain should prevent fraudulent activities and allow risk evaluation and countermeasures for its mitigation [23]. This requires tracing information. In addition, the traditional pen-and-paper-based SCM cannot properly monitor the process from production to distribution [24]. Therefore, food sector companies, and specifically aquaculture sector companies, have been required to implement smart traceability systems [25]. Nicolae et al. (2017) proposed an integrated traceability system for the fishery supply chain in Romania, which delivers wide-ranging, constant monitoring of food safety and quality at the national level [26]. It is also suggested that technologies such as blockchain, RFID, IoT, etc., can enhance the food SCM systems. For example, food contamination levels can be decreased by maintaining a certain level of temperature and humidity during the distribution process. These technologies are well accepted by consumers because of the visibility of the environment, ensuring the safety of supplies/products [27,28,29,30,31].

Currently, seafood consumers and importing countries have become more vigilant in assuring food safety and disease-free seafood/fish supplies. Even in this pandemic era, COVID-19 or other zoonotic diseases can readily spread if food safety is not adequately ensured. In July 2020, the demand for “*Chilean salmon*” dropped to “practically zero” in China after the cause of a COVID-19 outbreak was attributed to imported salmon. Experts believe that there is no evidence of virus transmission through food, even though Ecuadorian shrimp were also linked to a COVID-19 outbreak [32,33]. Nevertheless, food traders and buyers are still cautious about imported seafood products because food safety cannot be guaranteed regarding the use of agrochemicals and the introduction of genetic modifications (Genetically Modified Food). Food safety issues are related to the spread not only of COVID-19 but also of bird flu, foot-and-mouth disease, mad cow disease (Bovine Spongiform Encephalopathy, BSE), as well as to the possibility of inferior food floods [34,35]. Therefore, in 1997, a standard was formed by a group of retailers of the Euro-Retailer Produce Working group (EUREP) [36]. EUREP focused on food safety and the need for disease-free agricultural products and provided guidelines known as the “good agriculture practice (GAP)” and formed EUREPGAP, which was changed to GLOBALGAP in 2007 because of its global reach and its popularity in the world [37]. The United States (US) and Japan have also continuously implemented food traceability solutions to ensure safe consumption. Additionally, Australia, India, and China are in the process of establishing “food security” standards [38,39]. Therefore, it is essential to reinforce the safety measures for a risk-free food supply and increase consumers’ confidence [39]. As a result, the European Union subsidized the EU project “RFID from Farm to Fork” (RFID-F2F) to establish an internet-based RFID traceability system for the food and drink supply chain [40]. Consequently, various pilot projects developed in several fisheries and cold chain analysis sectors based on RFID and temperature monitoring sensors to relate environment conditions, product quality and safety [41,42].

This paper focuses on traceability solutions based on the RFID technology, which have been studied and implemented in many countries for fresh fish, aquaculture and seafood from production to processing to distribution. We also compared the outcomes of the use of this technology depending on its merits and demerits. This review article aims to ensure a traceability guideline for sustainability and safety in fishery producers, distributors, and consumers, so that the whole fishery SCM process will be automated, allow process monitoring from production to consumption and reduced the transaction and displacement costs.

## 2. RFID-Based Traceability Systems in Fishery SCM

The RFID technology has been widely used for traceability solutions, allowing safety, quality, and visibility to be maintained from production to consumption. Recent studies show that many countries have implemented RFID traceability solutions in the fishing industry to comply with quality requirements. RFID has been used in pilot experiments by researchers in fisheries SCM to ensure products’ trustworthiness in post-pandemic conditions. Additionally, the easy accessibility and mobility of the RFID technology make it popular worldwide for catch fish, live fish, aquaculture, and sea-food SCM systems. The following sections discuss RFID-based SCM traceability solutions for capture fish, aquaculture, aquatic products, and seafood implemented in various countries to collect, track data history, and allow access to information by the consumers throughout production, distribution, and selling processes.

### 2.1. RFID-Based Traceability Systems in Capture Fish SCM

Coronado Mondragon (2020) proposed a two-layer conceptual approach for the fishery sector. This research utilized a sensor layer based on WSN theory to model the surrounding energy consumption of a sensor network. In the first phase, data were collected from sensors used for ocean monitoring purposes. The collected data were analyzed using time series/scatter diagrams. As a result, the trends and patterns of snow crab catch settings were identified. Finally, this study presented a set of tools for future researchers in the fishery sector to develop this approach as monitoring mean for fisheries SCM with the help of IoT solutions, using the RFID technology [43].

Zhang et al. (2019) proposed an intelligent traceability platform based on the regulation of Hazard Analysis and Critical Control Points (HACCP). With this method, the wireless monitoring of facilities was integrated with quality control modeling to enhance the quality of fish and the safety and transparency of waterless fish transport. Therefore, a QR code and the RFID tag’s electronic product code (EPC) were combined to enable traceability functions to users for any query regarding tracking. In this way, results from a query regarding safe transportation were quickly provided to the consumers, from aquaculture to markets. In particular, sturgeon delivery trials were assessed and studied [44].

Kresna et al. (2017), developed an Internet Technology (IT)-based tuna traceability system for Indonesia, as this country is one of the leading tuna exporters, with a complex supply chain network. Due to its tendency towards high bacterial contamination (high Total Plate Count (TPC)), in particular by pathogens such as *Salmonella*, depending on the temperature and high content of histamine, a traceability system that could ensure the standard safety and quality of tuna SCM was mandatory. This traceability system ensures the safe handling, manufacturing, packaging, and transportation of tuna. This research prototyped a tracing system, illustrating the practical advantages of backward and forward tracing required for the tuna supply chain, from fishing vessels to retailers. Additionally, the system permits the biological examination of the products [45].

Treber et al. (2013) presented a RFID data logger for optimizing the supply chain system for perishable fish. Sensors with a RFID data logger were employed inside boxes to measure the temperature of products. Additionally, the RFID data logger was able to measure the temperature of the surrounding environment. The results found that the system was helpful to monitor the temperature of fish products during transportation. Moreover, using the mobile RFID reader, the sensor data were monitored in real time to ensure quality control, and the information was stored in the traceability system database. As a result, stakeholders and private consumers could access those data through the web [46].

Hsu et al. (2008) proposed a RFID-enabled SCM tracking system for live fish. In this research, the necessary information was collected for live fish processing, and plans were drawn up for the overall management system architecture, targeting small and medium enterprise solutions. In this method, a RFID tag was inserted into each live fish to monitor its movement in the living fish logistic centers and selling restaurants and provide information to the consumers for identification purposes. To collect the agribusiness-related information and for the automated transferring procedure, sensors were required, controlled by a PLC. Finally, a web-based solution was chosen in this research, which stored all the transferred tracking information of farmers and consumers. In this study, the overall system was implemented and arranged as a trial basis to collect valuable live fish information from logistic centers [47].

### 2.2. RFID-Based Traceability Systems in Aquaculture SCM

Abad et al. (2009) developed a real-time RFID smart tag for tracing and monitoring cold-chain food applications. A smart tag and a reader/writer were used in this procedure; the smart tag was attached to the products. These tags consisted of integrated lights, sensors (temperature and humidity), a memory to store product data, and an antenna for RFID tag communications. The memory chip stored the traceability data collected using the sensors. Following this, the research utilized a wireless reader to read the collected data of the food chain from a 10 cm distance with a mobility option. This method was able to read product data and track records automatically online and monitor the temperature of the cold chain. Additionally, this method does not require opening the polystyrene boxes containing the fish and the smart tag, so many tags can be read simultaneously when they pass through the fully automated reader. In addition, using temperature sensors, the system ensures that the temperature is kept below 0 °C for frozen foods. Humidity sensors are also present, making the system sensitive to humidity changes in the storage surroundings [48].

Kokkinos et al. (2018) developed an aquatic product traceability solution using IoT, RFID, and WSN. The solution integrated an internet platform, which was accessible via smart portable devices such as a RFID reader. The system was established to track and trace the security of aquatic products from their catch to the consumer’s table. Several wireless sensors were incorporated via the Arduino platform and RFID system. Fisheries’ conditions, different catching locations, and quality of fish products were maintained for sustainable fisheries. Additionally, depending on the conditions and quality assessment, protocols specific for the Greek sea, using classic and modern Artificial Intelligence (AI) methods, were presented [49].

Mira Treber (2015) proposed a RFID logistics processes management system for perishable food SCM. Real-time temperature monitoring management was included in the distribution process, which is the primary requirement of any cold chain system. She used RFID as the identification system for collecting information from production to shipment in cold storage. As a result, this pilot project successfully analyzed historical data monitoring the temperature with temperature sensors, which eventually increased the recall management systems’ efficiency [50].

Parreño-Marchante et al. (2014) presented a web-based traceability system that captures data utilizing the RFID system. The system integrates environmental data that are collected through wireless sensor networks (WSN) into that web-based service. Two pilot companies conducted this research in the aquaculture sector. The aim was to showcase how the overall business process in the aquaculture sector could be benefitted from and improved by this solution. The results found that by implementing the traceability solution, the studied companies achieved higher efficiency, up to 89–95%, along with activity time reduction. Therefore, key performance indicators (KPIs) were presented, including the reduction in activities time. However, the acceptance of electronic traceability systems for the food supply chain has not been as fast as expected [51].

Yan et al. (2012) designed and developed a traceable platform for aquatic food SCM employing RFID and EPC through IoT. They focused on developing an Object Name Service- (ONS) and EPC Information Service- (EPCIS) oriented platform for tracing, tracking, recalling, and monitoring *Tilapia* fish products for the SCM. Consumers, enterprises, and the government could benefit from this traceability solution. Additionally, the platform delivered a solution for aquaculture supervision, process management, distribution management, and sales management. Therefore, the traceability of aquatic food products was possible from breeding to processing, distribution, and sale [52].

Treber et al. (2011) highlighted two different examples of farmed fish tracking systems suitable for small- and medium-sized enterprises (SMEs). The first one involved changing a manual data collection method to an electronic RFID-enabled scheme implemented in a small company. This project realized a complete SCM solution for farm fish useful to selling agencies and private customers. The second solution consisted of handling part of the automated process of packing fish labeled with a barcode, upgraded by the RFID technology. In this case, the aim was to extend the traceability to breeding and on-growing fish farms from a manual data collection process to a RFID-enabled data collection method. The pilot implementation was based on modules that used few mobile RFID readers in different steps of the process. These modules intend to use a general approach for an automated business procedure [53].

### 2.3. RFID Traceability for Seafood SCM

The seafood traceability system proposed by Wang et al. (2016) combined advanced technologies such as QRcode, RFID, and barcode with image processing. The system was able to improve the efficiency and accuracy of operation and information collection for seafood traceability. A full solution of seafood traceability from breeding to processing and sale logistics was presented by Wang et al. [54].

Mai et al. (2010) performed cost–benefit analysis on the fish supply chain in 2010 using a RFID-based traceability solution. They aimed to conduct a study of two firms using different operating steps of the seafood supply chain. In this research, the aim was also to obtain preliminary knowledge regarding the cost–benefits for the project actors and to describe the tangible, quantifiable benefits of implementing RFID traceability solutions for seafood trading companies. Additionally, the authors suggested that the RFID tracking implementation costs should be borne by the firms and highlighted the benefits of using this RFID-based solution as a future marketing tool according to food safety regulations [55].

To analyze the distribution channel of marine products in Korea, Park et el. (2007) redesigned the marine distribution channel using RFID technology. They performed a case study on seafood SCM in Korea and presented a comprehensive solution for managing the whole seafood distribution channel. Additionally, a RFID-based ubiquitous environment was suggested to allow far more efficient control planning for the seafood distribution channel in addition to the expected effects [56].

## 3. Discussion

Modern supply chains have evolved from allowing product tracing and tracking to enabling the monitoring of highly complex networks, using connections between environment and machine, machine and machine, and machine and human. Technology has provided advantages to suppliers, distributors, manufacturers, and retailers. However, it is challenging to obtain an efficient and versatile SCM system. This system is expected to verify raw materials, store products in a limited space in the inventory, maintain the visibility of the product along the chain, and enhance the customer experience in retail shops. This research highlights numerous challenges encountered in recent studies. A RFID–IoT system includes RFID and IoT technology, emphasizing the connection of all components through sensors, cheaper processors, and ubiquitous computing. The desire is to improve the hardware aspect of the technology, increase its reliability, and minimize the deficiencies during product tracing and tracking. A comparison of recent research based on the results and target applications in the fish industry is presented in Table 1, Table 2 and Table 3.

Table 1 presents a summary of RFID traceability solutions for different capture fish SCM systems, which have been implemented globally. Though different sectors chose to implement traceability solutions using the RFID technology in different countries, most studies were expanded their pilot projects to real-time applications for catch fish SCM systems. Table 1 illustrates that RFID-based traceability solutions have been implemented for several categories of catch or fresh fish, from production to consumptions (fresh fish, live fish, cod, or tuna fish). These results indicate that RFID-based applications in catch fish SCM systems have provided various benefits, such as temperature alerts, visibility from production to sale, conventional traceability tools, no requirement to engage the human workforce, reduced contamination levels, and, most importantly, the possibility to read the information on many tags at the same time, thus maximizing business opportunities [43,44,45,46,47]. However, a few drawbacks have been found for these proposed systems. In the case of fresh fish traceability, the involvement of the blockchain would provide higher reliability of the collected information [43]. In addition, unstable and imprecise live fish survivability monitoring and controlling has been observed [44]. In addition, some case studies reported only the pilot project’s outcomes and need to test the feasibility of large-scale implementation [45]. Several studies concluded that shelf-life calculations and the assessment of critical measurements are necessary [46]. Another issue is that RFID tags can easily be damaged when used in live fish or fresh fish, and radio frequency signals can be lost due to close contact with the water [47].

**Table 2:** Comparison of RFID traceability solutions for Aquaculture SCM systems.

Technology	SCM	Advantage	Disadvantage	Reference
RFID Tag	Cold Chain	- Does not require opening the polystyrene boxes containing the fish and the smart tag - Many tags can be read simultaneously when they pass through the fully automated reader	- Required commercial credit card-like packaging to integrate the smart tags in the polystyrene boxes	[48]
RFID and WSN	Aquatic Products	- Can predict the product lifeline - Able to calculate the just-in-time inventory of outlets - Minimize the waiting time up to consumption	- Lack of consumers, transporters, and sellers’ interest in using the smart devices - Did not compare the corresponding evaluation procedures for cold chain supply	[49]
RFID logistics	Cold Chain	- -Increased recall management systems efficiency	- The movement speed is crucial for tracing the box information	[50]
RFID and WSN	Aquaculture	- -Increase the consumer belief through enhancements in product regulation, groups, supervision of time, and automated process. - Higher efficiency	- The tests were performed for a limited period - Additional tests are required to gather more information	[51]
RFID and EPC	Aquatic Foods	- A solution for aquaculture supervision, process management, distribution management, and sales management	- High implantation cost	[52]
RFID	Farm Fish	- Proposed a flexible, scalable, and interoperable system for traceability - Easily transferrable to farmed fish-based business methods	- The results are limited to the specified cases and conditions	[53]

Table 2 compares different SCM traceability systems for aquaculture or aquatic products using the RFID technology. RFID-based traceability for aquaculture SCM systems has increased the consumer belief through enhancements in product regulation, groups, supervision of time, and automated processes. Moreover, an easily traceable solution of the farmed business SCM is maintained by offering a supple, scalable, and interoperable structure. Previous researchers have implemented RFID trackability for cold chain, aquatic products, and farmed fish [48,49,50,51,52,53]. Cold chain temperature and humidity are maintained by employing RFID technology in this sector [50]. However, consumers, transporters, and sellers have shown little interest in using the smart RFID devices [48]. In addition, this smart solution did not compare the corresponding evaluation procedures for cold chain supply, which can be easily transferrable to farmed fish-based business methods [53]. Additionally, the high implantation cost and the limited outputs for specific scenarios are drawbacks of the solutions proposed by Yan et al. (2012) and Mai et al. (2010).

**Table 3:** Comparison of RFID traceability solutions for Seafood SCM systems.

Technology	SCM	Advantage	Disadvantage	References
QRCode, RFID and image processing	Seafood	- Improve the efficiency and accuracy of operation and data collection	- Complex system; requires several technologies at a time - Low-quality images may reduce traceability accuracy	[54]
RFID	Seafood	- Provides a solution to retain existing customers, improves product quality, and reduces consumer complaints - Provides a cost-benefit analysis	- No portable terminals for operations - Lack of packaging; no need to integrate smart tags in the polystyrene boxes	[55]
RFID	Seafood	- Suggests an effective foundation for seafood SCM - Provides a whole management solution for distribution channels	- Large distributors are necessary for the system to be efficient	[56]

Table 3 compares RFID-based traceability for seafood SCM systems. By implementing this technology, product quality, transportation efficiency, and accuracy have been improved [54,55,56]. Additionally, the RFID technology has allowed for retaining existing customers and reducing consumer complaints [54]. A cost–benefit analysis was also provided in a few cases [55], which suggested that seafood SCM is promising for the whole distribution channel [56]. However, few demerits have been identified from these studies, e.g., portable terminals for operations would provide a better solution for seafood SCM. In addition, Abad et al. (2009) did not propose any packaging or integration of smart tags in polystyrene boxes in the RFID-based SCM. Additionally, a large number of distributors are required for the solution presented by Park et al. (2007).

RFID in SCM also highlights the limitations regarding business information and trace data. It would be an effective and simplified solution for quick content analysis by future fish consumers. Moreover, using RFID-based traceability systems for future SCM will protect track data from fraud, exploitation, alteration, falsification, etc. [57,58].

At present, RFID is one of the wireless technologies most widely used in many countries to track product information remotely with the help of IoT. It is possible to trace and monitor the temperature for frozen fish logistic chains [59,60]. Moreover, maintaining a specific level of humidity is necessary for fresh fish and seafood during the storage process. Because of the ability to sense humidity, RFID can be used more ubiquitously in the fishery industry. The feature of real-time monitoring and validation makes it a convenient application in the fresh fish logistic chain. Moreover, it uses the automated system of tracking based on RFID tags and reader data and integrates the information into an online database in real time. This system can read RFID tag data of fresh fish, seafood, frozen food, or even canned food without packaging boxes (polystyrene boxes, Styrofoam boxes) [61]. It also reads many RFID tags simultaneously, automatically passing the information to the reader. It is possible to obtain real-time traceability information from production to consumption with the different food/fish SCM systems. As a result, safety and quality are maintained along fisheries’ logistic chains; this improves the SCM and strengthens consumers’ confidence in the fish/seafood logistic chain.

To improve resources and costs, there is a need to further analyze the collected data. Although several studies have analyzed RFID data processing and sharing solutions in the supply chain, the cost of computing and the efficiency of storage are usually not considered [62]. Fortunately, with the emergence of cloud technology, we have an accessible platform for on-demand computing microservices, especially for data exchange and integration with other systems at a low cost. Instead of developing their system infrastructure from scratch, developers “rent” a technology from a cloud service provider. This helps decrease the cost of software and hardware development and of human resources. Furthermore, the developer can also reduce the high maintenance fees as the cloud provider handles many services which can be customized based on the requirements. Additionally, a cloud service provider such as the Google Cloud Platform offers a “pay-as-you-go” solution without any up-front and termination fees [63]. This process has encouraged the fast development of proof-of-concept studies with minimal cost and use of resources [64].

In the future, we look forward to more research into innovative SCM processes using RFID tags and readers with higher sensitivity, feasibility, and adaptability. Transmission of large amounts of data can cause system delay, communication errors, and conflicts between the retailers and the consumers. The challenge is even more complicated when most of the data are collected in real time by connected devices. Once data are collected, the process of analyzing them, including data validation, cleaning, mining, exploring, and loading, requires highly skilled workers and powerful computing hardware such as a Computer Processing Unit (CPU) or Graphic Processing Unit (GPU). Furthermore, the hardware can be designed in miniature, with low energy consumption, embedded, and is easy to implement with other technologies. Besides that, the lack of seamless integration between the current existing system and the newly developed open-source-based system would be an issue of study. Many proposed systems are used in large-scale supply chain productions in the fishery sector. However, the maintenance of the system pipeline, network architecture, and data management is complex and may require a new design of the whole architecture. At the same time, with the increasing development of RFID devices, it remains a significant challenge to develop a fishery SCM system infrastructure that can manage massive data within the same network.

## 4. Conclusions

In this review article, we discussed the traceability systems of sustainable and safe fisheries SCM using the RFID technology. Due to the advantages of the RFID technology, we found that most pilot studies led to real-time implementation in fresh fish and aquaculture SCM systems. However, the RFID technology has been applied only to a limited extent for fisheries or seafood traceability compared to other food industries. The findings from the literature review show that most of the systems aimed to implement a solution with a sensing capability that allows information transfer through the RFID technology. The use of the RFID technology in fisheries SCM is required to regulate food safety and quality. However, the system’s current operation relies on multiple technologies, which increases the cost of its development. RFID-based aquaculture or seafood traceability in SCM will also resolve potential technological issues such as customer requests and fast changes in orders. In addition, the cost of operation and adoption of new systems is increased by the incompatibility with existing systems. A smooth and feasible project workflow with coordinated efforts is needed to ensure the efficiency of fisheries SCM while controlling the overall cost of RFID-based systems. This review explored the rise of sustainable and safe fisheries traceability systems based on RFID technology and consumer’s acceptability in SCM. Through this technology, fisheries’ SCM will be able to evaluate the achievable profit, ensuring a sustainable, safe supply and maintaining proper storage conditions, temperature, and humidity, and to compare the advantages with the cost of implementing the system, to make a wise decision.

## Figures and Tables

**Table 1:** Comparison of RFID traceability solutions for the Capture Fish SCM system.

Technology	SCM	Advantage	Disadvantage	Reference
WSN and RFID	Fresh Fish	- Promote the visibility of the supply chain without line-of-sight scanning process - Reduces excessive workforce cost but also maximizes business opportunities	- Blockchain technology involvement is required for higher reliability of the information	[43]
RFID and WSN	Waterless Live fish	- Reduced contamination level and risks by maintaining the proper temperature. - Provide a quality control solution with low implementation cost and high endurance outcome - -Enhance effectiveness through proper technical references	- Waterless live fish transportation is still at the primary stage - Unstable and imprecise live fish survivability monitoring and controlling	[44]
IoT and RFID	Tuna supply chain	- Permit the monitoring of methods and goods based on microbiological investigation, and current SOPs are preserved	- A prototype, which was not implemented in real-time application	[45]
RFID with Sensors	Fresh Fish	- Ensure quality control - Ensure temperature - Data can be accessed through the web	- Need shelf-life calculations - Need to examine and recognize critical measurements	[46]
RFID	Live fish logistic chain	- No engagement of the human workforce is required. - Able to read many tags at the same time without tag visibility - More flexible technology in terms of humidity and ecological conditions	- RFID tag destroyed due to harmful fish species - Limited radio frequency signal caused by the water	[47]

## Data Availability

Not Applicable.
